# Graft-derived VWF drives platelet activation and thrombocytopenia during porcine liver xenotransplantation to brain-dead human recipients

**DOI:** 10.1172/JCI200800

**Published:** 2026-03-10

**Authors:** Liang Zhao, Sokratis A. Apostolidis, Aae Suzuki, Amrita Sarkar, Qian Guo, Felix Li, Alex Sagar, John Fallon, Mohamed A. Elzawahry, Syed Hussain Abbas, Leanne Lanieri, Kristen Getchell, Susan C. Low, Kim M. Olthoff, Emma E. Furth, Brendan J. Keating, Peter Friend, Mortimer Poncz, Abraham Shaked, Charles S. Abrams

**Affiliations:** 1 Department of Medicine, Perelman School of Medicine, University of Pennsylvania, Philadelphia, Pennsylvania, USA.; 2 Children’s Hospital of Philadelphia, Philadelphia, Pennsylvania, USA.; 3 Division of Transplantation, Department of Surgery, New York University Langone Health, New York, New York, USA.; 4 Nuffield Department of Surgical Sciences, University of Oxford, The Churchill Hospital, Oxford, United Kingdom.; 5 eGenesis, Cambridge, Massachusetts, USA.; 6 Penn Transplant Institute, Hospital of the University of Pennsylvania, Philadelphia, Pennsylvania, USA.; 7 Department of Pathology and Laboratory Medicine, Perelman School of Medicine, University of Pennsylvania, Philadelphia, Pennsylvania, USA.

**Keywords:** Hematology, Vascular biology, Transplantation

## Abstract

**BACKGROUND:**

Genetically engineered porcine livers are being developed as a bridge therapy for acute liver failure, providing detoxification and restoration of hepatic protein synthesis. Severe xenograft-associated thrombocytopenia remains a major limitation, and human mechanistic data are scarce.

**METHODS:**

Platelet kinetics were characterized in 3 human decedents undergoing extracorporeal cross-circulation with transgenic porcine livers. Platelet counts, transfusion requirements, and clearance patterns were assessed to distinguish consumption from marrow suppression or hypersplenism. Antibody- and complement-directed inhibitors were administered to test immune-mediated mechanisms. Mechanistic studies focused on porcine von Willebrand factor–dependent (pVWF-dependent) platelet activation, including ex vivo blockade with the anti-VWF nanobody caplacizumab, a VWF-directed antibody fragment that prevents VWF-platelet binding. A fourth decedent received caplacizumab during porcine liver perfusion.

**RESULTS:**

In all 3 initial cases, 80%–90% of circulating and transfused platelets were rapidly cleared, a pattern inconsistent with marrow suppression or hypersplenism. Antibody and complement inhibition failed to ameliorate thrombocytopenia. Recipient plasma induced robust pVWF-mediated platelet activation analogous to human type IIb von Willebrand disease, which was completely abrogated ex vivo by caplacizumab. In a fourth decedent treated with caplacizumab, aberrant platelet activation was prevented, although full hematologic recovery was limited by preexisting disseminated intravascular coagulation.

**CONCLUSIONS:**

Early thrombocytopenia during porcine liver xenotransplantation appears to be primarily driven by pVWF-mediated platelet activation rather than by classical immune or splenic mechanisms. Targeted VWF blockade with agents such as caplacizumab may mitigate platelet loss and improve the safety profile of extracorporeal porcine liver support in acute liver failure.

## Abstract 2

Genetically engineered porcine liver xenotransplants cause severe platelet loss mediated by porcine Von Willebrand Factor. Targeted VWF inhibition is a potential therapeutic strategy.

## Graphical abstract

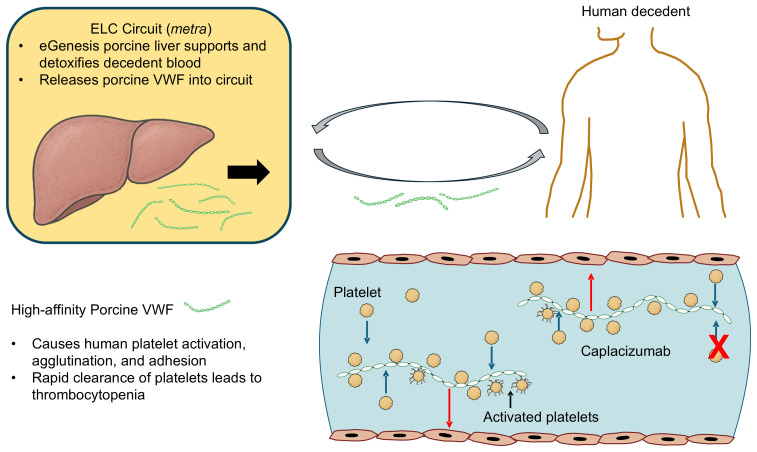

## Introduction

Liver failure, whether acute or acute-on-chronic, severely disrupts hepatic, metabolic, and synthetic functions, leading to a dangerous buildup of toxins and widespread physiological collapse. Liver failure carries a 30%–40% mortality rate within 3 to 4 weeks, and therapeutic options remain limited (1–3). Among the clinically consequential manifestations is a severe coagulopathy, arising from impaired production of clotting factors combined with systemic inflammation and consumptive processes. Initial supportive measures may permit native liver recovery or act as a bridge to transplantation. Previous studies have tested artificial extracorporeal devices for temporary patient stabilization and partial blood detoxification. However, despite extensive evaluation, these methods have not demonstrated a clear survival benefit (4–9). The need for donor organs remains constant, driven by an aging population and the increasing incidence of liver disease.

Xenotransplantation offers a transformative solution to temporary stabilization and reversal of acute liver failure. Genetically engineered porcine livers metabolize toxins and provide synthetic support by synthesizing coagulation factors and other essential proteins that could sustain patient recovery until a compatible human donor organ becomes available (10–13). Recent application of CRISPR-based gene editing has enhanced the feasibility of successful xenogenic liver support (14). Specific genetic modifications of porcine xenografts have minimized immunological incompatibilities and pathogen transmission, thereby positioning xenotransplantation as a practical and scalable therapeutic option to bridge critical periods of hepatic insufficiency (11, 13, 15–18). For patients with acute or acute-on-chronic liver failure, the prospect of ready access to compatible and functioning grafts could dramatically lower waitlist mortality and enable timely intervention for those currently deemed transplant ineligible due to disease acuity or lack of donor organ availability (19, 20). As these technologies and genetic modifications advance, hepatic xenotransplantation could potentially revolutionize care for end-stage liver failure (15).

**Figure 1:**
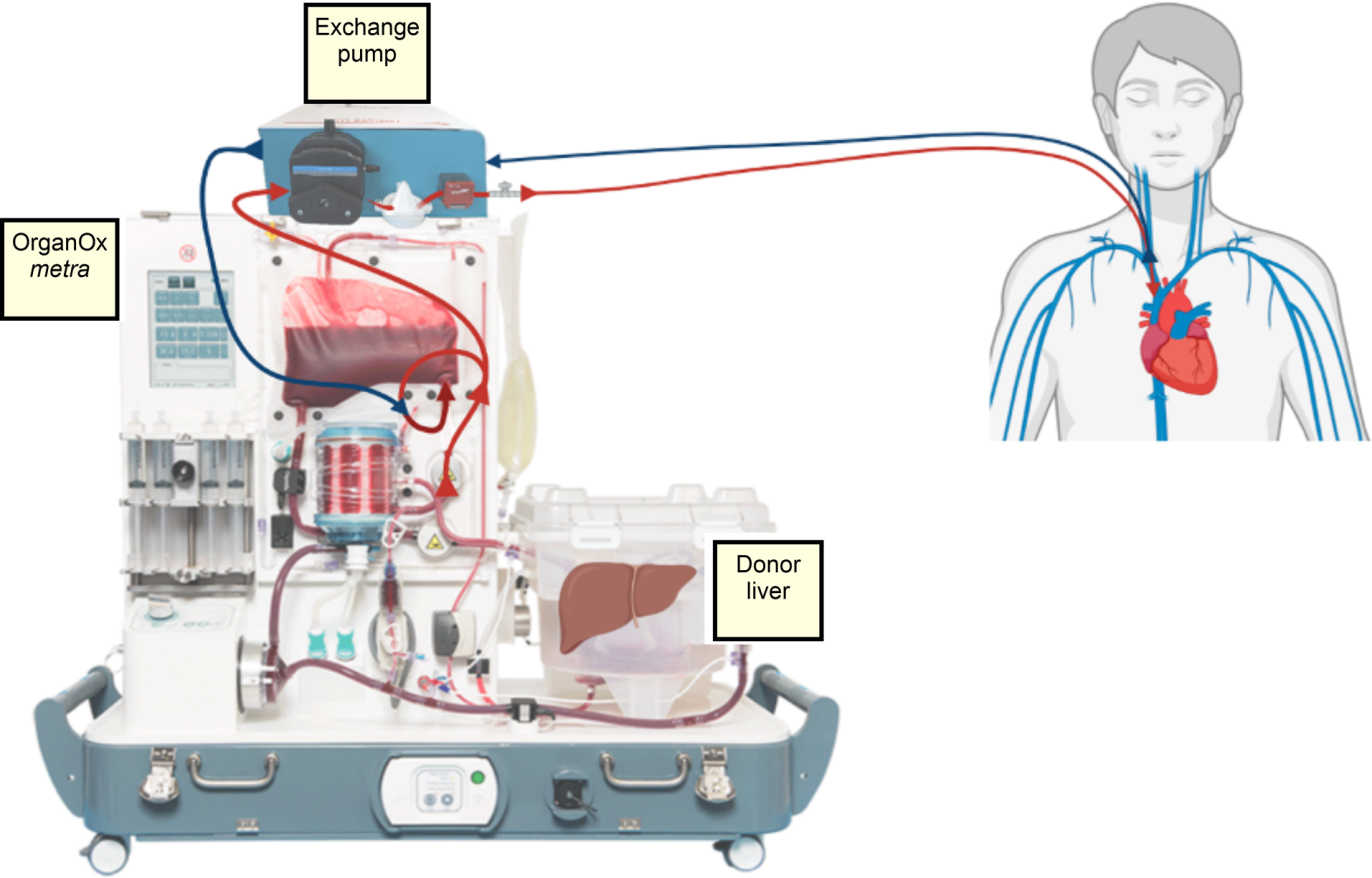
Schematic of extracorporeal cross-circuit. Extracorporeal cross-circulation in which blood from a human participant is continuously circulated through a closed perfusion circuit. The porcine liver is supported within a normothermic machine oxygenator that preserves its viability and enables physiologic, metabolic, and immune interactions during cross-circulation. As shown in the schematic, blood flows from the patient to the *metra* device where it is oxygenated and perfused through the porcine liver prior to reinfusion back into the patient.

Genetically modified porcine livers are also being explored for short-term hepatic support using extracorporeal liver cross-circulation, a method where a donor liver is maintained outside the body using a normothermic machine perfusion that is connected to the bloodstream of the patient via an exchange pump (Figure 1) (18, 21–23). This approach provides metabolic support and restores synthetic function, including correction of the coagulopathy of liver failure, while the native liver regenerates or until a transplant becomes available. Extracorporeal liver cross-circulation aims to bridge critical gaps in the treatment for acute/acute-on-chronic liver failure overcoming limitations of current therapies, including organ shortages and the inadequate hepatic support provided by currently available supportive technologies.

While experimental transplants in nonhuman primates and early human studies have shown some success, a major obstacle to liver xenotransplantation in nonhuman primates has been the development of profound thrombocytopenia and bleeding that rapidly ensues following the transplant of porcine livers, which at times leads to recipient death (24, 25). The cause of the thrombocytopenia in nonhuman primates has been controversial. It is speculated to involve porcine hepatic endothelial cell–mediated phagocytosis, spontaneous agglutination of human platelets by porcine von Willebrand factor (pVWF), and asialoglycoprotein-dependent phagocytosis of platelets by porcine Kupffer cells (26–30).

In addition to thrombocytopenia, concern about coagulation disturbances is another potential barrier to successful liver xenotransplantation (31). Cross-species incompatibilities in key hemostatic factors that include pVWF, tissue factor expression, and complement regulatory proteins could create imbalances in coagulation activation and clearance (28, 32). Preformed natural antibodies directed against carbohydrate xenoantigens (e.g., α-Gal, Neu5Gc) might also initiate complement activation and endothelial injury, amplifying thrombotic and inflammatory cascades (16). Despite advances in genetically engineered pigs with targeted knockouts and humanized transgenes that reduce antibody binding and complement activation, residual incompatibilities remain (11). These immunologic and molecular mismatches underscore the delicate balance between thrombosis and hemorrhage that complicates porcine liver xenograft survival and recipient safety (12, 31).

Brain-dead human decedents offer a controlled environment to study physiological responses to porcine liver xenografts, particularly during the critical early posttransplant phase (33–36). This model reliably replicates immune, metabolic, and pharmacological interactions specific to human recipients, thereby bypassing the limitations of animal studies. These investigations validate preclinical findings and establish clinical proof of concept for extracorporeal liver cross-circulation using transgenic porcine grafts in acute liver failure. In this study, we utilized this model to elucidate the mechanisms underlying hematologic dysregulation observed after extracorporeal liver cross-circulation with a porcine liver.

## Results

**Figure 2:**
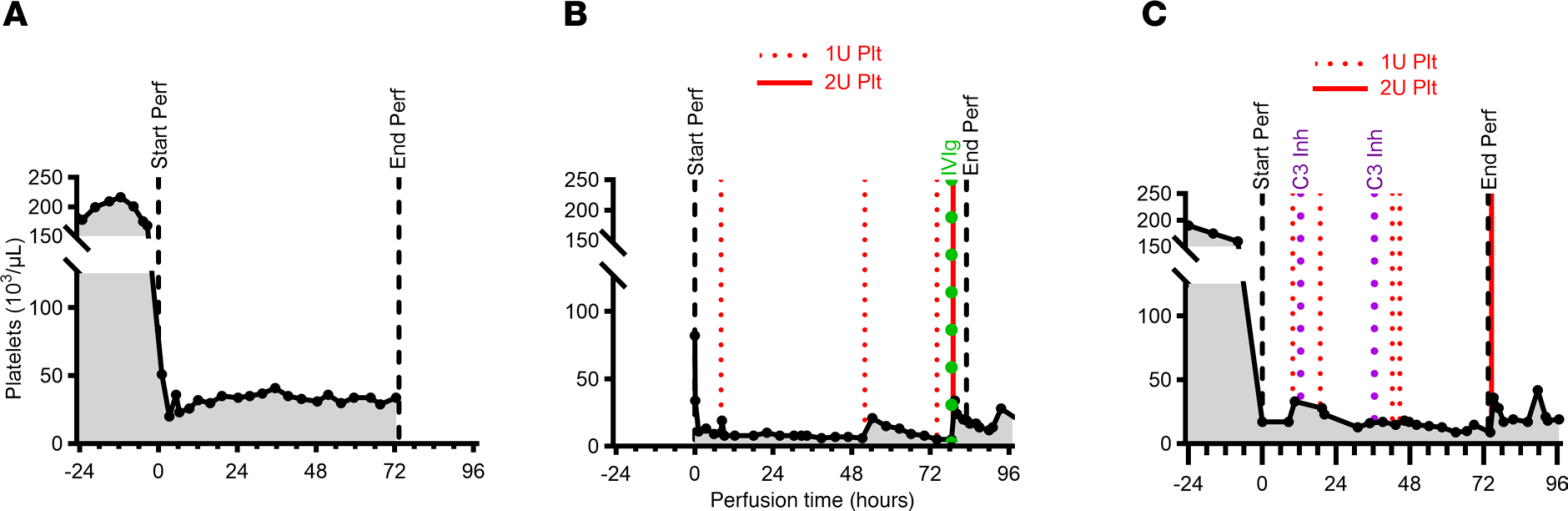
Platelet counts in decedents receiving extracorporeal liver cross-circulation with porcine livers. (**A**–**C**) Serial platelet counts of Decedents 1–3 before, during, and after disconnection from extracorporeal liver cross-circulation. Red lines: infusion of platelets. IVIg: infusion of immunoglobulin. C3 inh: infusion of pegcetacoplan C3 inhibitor.

At the outset of our study, we assessed platelet dynamics during extracorporeal cross-circulation of transgenic porcine (EGEN-5784) livers with the initial 3 brain-dead recipients. In all cases, thrombocytopenia developed with remarkable speed, with 80%–90% of circulating platelets (both endogenous and transfused allogeneic) cleared from the bloodstream in less than 1 hour (Figure 2, A–C). The thrombocytopenia in all these decedents developed too rapidly to be attributed to a bone marrow megakaryopoiesis defect and was too severe to result solely from hypersplenism. Previous observations in nonhuman primates receiving a different transgenic porcine liver (Gal-only genetic deletion) xenotransplant also noted thrombocytopenia. This was hypothesized to occur via porcine endothelial cells phagocytosing platelets (29). However, the extremely rapid platelet clearance seen in primate recipients and in these 3 decedents challenges whether nonprofessional phagocytes (like endothelial cells) possess sufficient phagocytic efficiency to explain such acute thrombocytopenia in vivo. This accelerated onset of thrombocytopenia narrows the potential mechanisms of low platelet counts in xenotransplantation to (a) antibody-mediated platelet phagocytosis by the Fc receptor–expressing macrophages in the spleen, (b) a complement-driven process that induces platelet lysis or phagocytosis of C3-bound platelets by C3 receptor–expressing liver macrophages, or (c) molecules such as VWF that induce platelet agglutination, adhesion, or aggregation, and result in platelet clearance within the liver or spleen. These potential mechanisms were analyzed individually.

We hypothesized that during extracorporeal liver cross-circulation connection to the porcine liver, human platelet surface proteins might be modified in a manner that permits the binding of preexisting recipient antibodies and promotes Fc receptor–mediated clearance by splenic macrophages. However, as shown in Figure 2B, administration of intravenous human immunoglobulin (IVIg) to Decedent 2 during extracorporeal liver cross-circulation had little to no effect on platelet clearance. Moreover, even if porcine B cells or plasma cells within the liver could produce anti–human platelet antibodies, the time interval was insufficient for de novo antibody generation capable of mediating the rapid destruction of human platelets. These findings indicate that antibody-mediated mechanisms were unlikely to account for the observed thrombocytopenia.

Complement activation in the decedents could plausibly contribute to thrombocytopenia through 2 mechanisms: intravascular platelet lysis, analogous to paroxysmal nocturnal hemoglobinuria (PNH), or phagocytosis of C3-opsonized platelets by the C3 receptor–expressing macrophages in either the native or porcine liver. To evaluate the possibility of complement-mediated thrombocytopenia, we administered repeated doses of pegcetacoplan (an FDA-approved C3 inhibitor effective in blocking both complement pathways in PNH) to Decedent 3 (37). The pegcetacoplan was given while the decedent was profoundly thrombocytopenic at approximately 12 hours after connection to the porcine liver, and a second dose of pegcetacoplan was administered approximately 24 hours later. Despite treatment, the decedent remained profoundly thrombocytopenic and refractory to platelet transfusions at all time points (Figure 2C), demonstrating no improvement in transfused platelet survival. These findings indicate that neither complement-mediated lysis nor phagocytosis of C3-opsonized platelets is a principal mechanism underlying platelet loss in this setting. The experiments using IVIg and pegcetacoplan argued that the thrombocytopenia was not entirely immune mediated and suggested that other factors may be responsible for platelet clearance following extracorporeal liver cross-circulation with porcine livers such as pVWF.

### Porcine-derived proteins induce platelet agglutination, aggregation, and adhesion.

**Figure 3:**
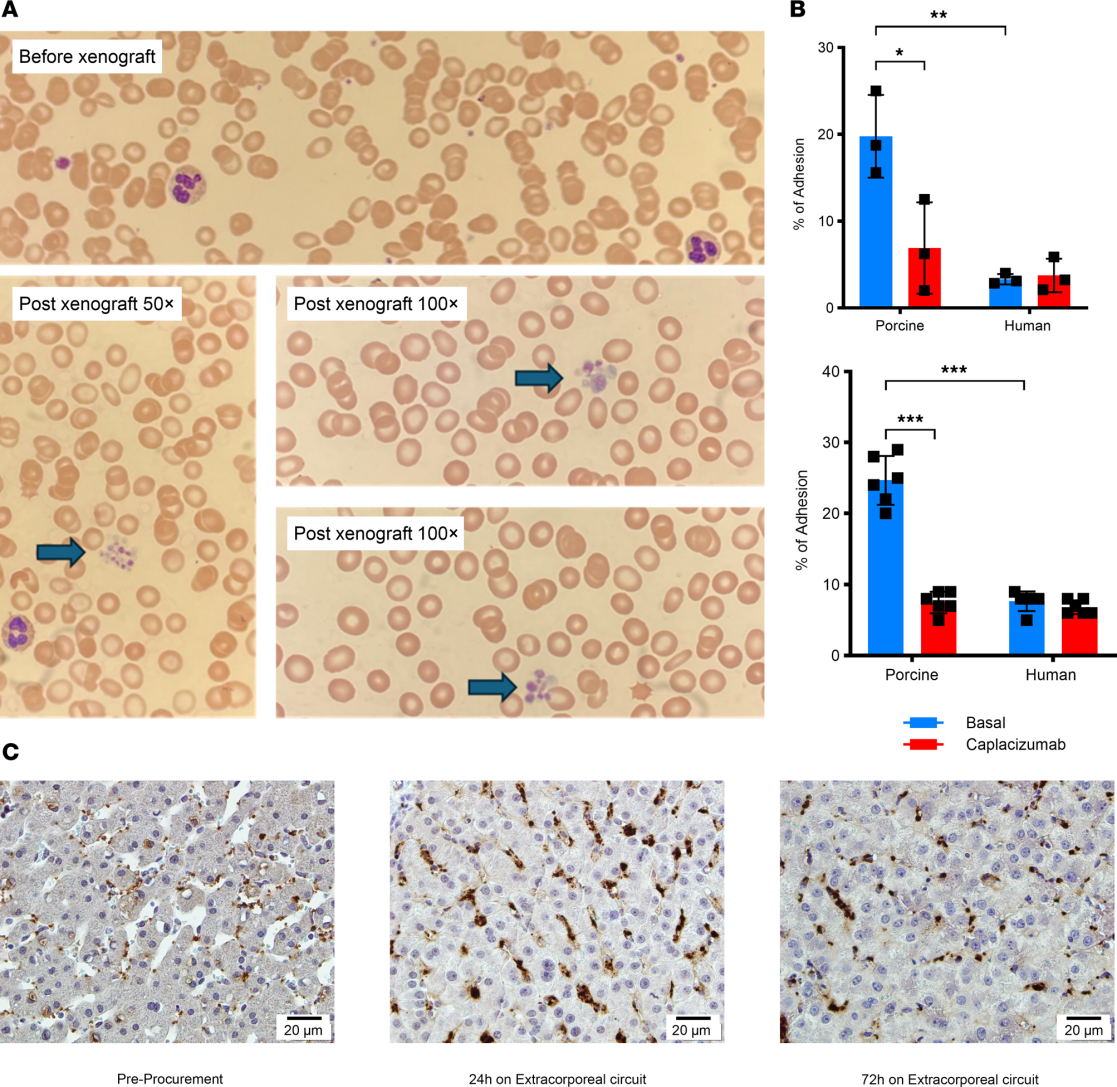
Thrombocytopenia and platelet clumping exhibited in decedent receiving extracorporeal liver cross-circulation with porcine liver. (**A**) The peripheral blood smears showing ×50 magnification of the peripheral blood smears from Decedent 3 before or after connection to the xenograft. After connection to the xenograft, most platelets in Decedent 3 were dispersed and separate, although small clusters of platelets (indicated by arrows) could be occasionally seen at both ×50 and ×100 magnification. (**B**) Spontaneous adhesion of washed human platelets to porcine and human endothelial cells is demonstrated under static (top panel) and flow (bottom panel) conditions. The presence of an anti-VWF nanobody caplacizumab significantly attenuated the spontaneous adhesion of human platelets to porcine endothelial cells. *P < 0.05; **P < 0.01; ***P < 0.001. (**C**) Platelet accumulation was observed in the porcine liver following the connection to Decedent 1. Immunohistochemical staining for platelet integrin (CD41) was performed on porcine liver samples obtained before and after connection. Scale bars: 20 μm.

Examination of the peripheral blood smears confirmed the thrombocytopenia in the decedents, with notable absence of schistocytes or nucleated red blood cells in Decedents 1–3, which rules out microangiopathic processes. Although most platelets appeared isolated, rare small platelet clusters were observed (Figure 3A). This is distinct from pseudothrombocytopenia patterns due to their scarcity and size. These small clusters of platelets were not observed on peripheral blood smears of the decedents prior to their extracorporeal liver cross-circulation with porcine livers. Given these findings, we investigated whether a platelet-binding molecule could mediate thrombocytopenia through an adhesion, aggregation, or agglutination mechanism that would lead to hepatic or splenic clearance.

One potential mediator of platelet agglutination is pVWF, which possesses a unique glycosylation profile and a deletion within a regulatory region, both of which have been postulated to enhance its ability to spontaneously bind to the human platelet GPIb receptor (38, 39). This property closely parallels type IIb VWF mutations in humans, which similarly promote spontaneous GPIb engagement (40). In this context, presynthesized pVWF would be stored in Weibel-Palade bodies within hepatic porcine endothelial cells and upon exocytosis serve as the source of pVWF in the bloodstream (41, 42).

If spontaneous binding of pVWF to human platelets occurs during extracorporeal liver cross-circulation with porcine livers, this could enable direct platelet adhesion to the hepatic vasculature. Such interactions would be expected to activate platelets, leading to the release of ADP and thromboxane A2. The resulting signaling cascade would drive rapid platelet aggregation and agglutinate formation, which could subsequently be sequestered and cleared in the spleen, liver, or lungs. We found that human platelets spontaneously adhered to cultured porcine, but not to human, endothelial cells (Figure 3B), which is consistent with the proposed mechanism of VWF from porcine endothelial cells binding and agglutinating human platelets. In support of this model, platelet-porcine endothelial adhesion was abolished by an inhibitor targeting VWF, demonstrating that this interaction depends on VWF. Immunohistochemical staining for the platelet-specific antigen CD41 was performed on porcine liver samples obtained before and after connection to Decedent 1. While some isolated porcine platelets were detectable prior to organ procurement, CD41 staining increased markedly during perfusion through the extracorporeal circuit, indicating the accumulation of human platelets within the porcine liver (Figure 3C).

### Effect of VWF inhibition on xenograft induced thrombocytopenia and platelet agglutination.

Under physiologic conditions, VWF does not bind to circulating platelets. Binding occurs only after vascular injury exposes subendothelial collagen, where VWF adheres and undergoes shear stress–induced conformational changes. This exposes the cryptic A1 domain, enabling binding to platelet receptor CD42 (39). In type IIb von Willebrand disease (VWD), a gain-of-function VWF mutant binds spontaneously and excessively to platelets without inducing vascular injury. This causes intravascular platelet agglutination, activation, and thrombocytopenia due to the clearance of VWF-platelet complexes (40). Diagnosis of type IIb VWD can be accomplished by using the ristocetin-induced platelet agglutination (RIPA) test. Ristocetin is a glycopeptide antibiotic that enhances VWF-platelet binding. Type IIb VWD is confirmed when a patient’s VWF causes platelet agglutination at lower ristocetin concentrations than required by normal VWF (43). By analogy, we hypothesize that pVWF released from porcine livers may contribute to thrombocytopenia in decedents connected to porcine livers via this same pathological mechanism (44–46).

**Figure 4:**
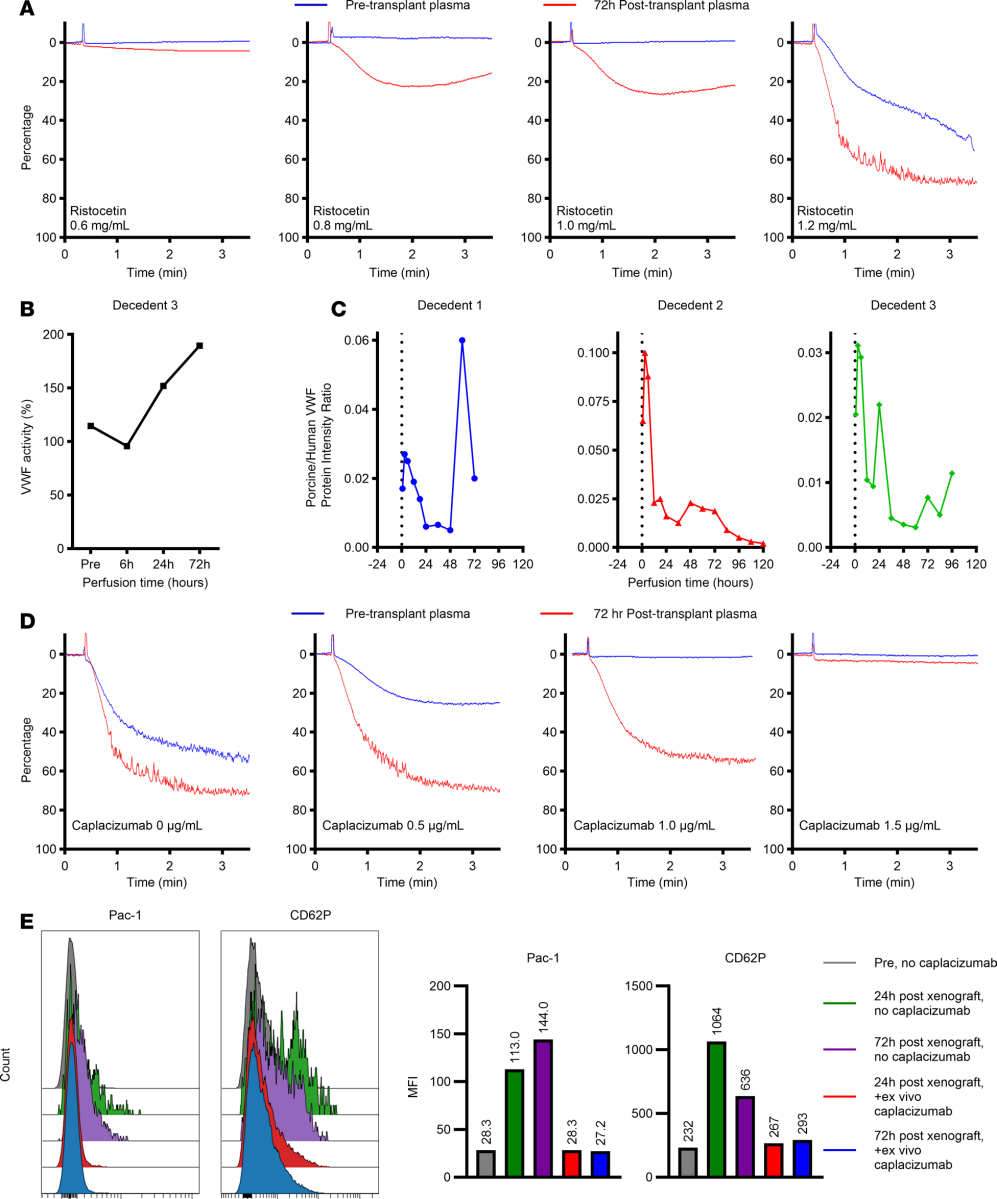
Analysis of VWF during post-extracorporeal liver cross-circulation. (**A**) Shown are ristocetin-induced platelet agglutination (RIPA) assays. Washed human platelets isolated from healthy donors were exposed to plasma derived from Decedent 3 at various time points before and after extracorporeal liver cross-circulation with a porcine liver. Following the addition of different concentrations of ristocetin, plasma obtained 72 hours after connection to the porcine liver induced increased platelet agglutination compared with plasma obtained from the decedent prior to exposure to the porcine liver. (**B**) VWF functional activity assays performed before connection and at indicated time points after connection to the porcine xenograft. (**C**) Mass spectrometry of decedent plasma demonstrates the relative amounts of both porcine and human VWF after the start of extracorporeal liver cross-circulation. (**D**) Compared with plasma obtained from a decedent prior to extracorporeal liver cross-circulation with the porcine liver, plasma obtained from a decedent after the start of extracorporeal liver cross-circulation with porcine liver requires higher concentrations of caplacizumab to neutralize the ability of VWF to agglutinate human platelets. (**E**) Plasma from Decedent 3 was added ex vivo to healthy human control platelets, and flow cytometry was used to analyze for platelet activation as detected by the binding of the PAC-1 (activated integrin) or the CD62P (P-selectin) antibody. Shown on the left are histogram plots and on the right is a bar graph demonstrating the relative PAC-1 and CD62P surface expression on ex vivo–isolated platelets at the indicated time points and conditions. Note that caplacizumab abolished plasma-induced platelet activation when administered ex vivo.

As demonstrated in Figure 4A, plasma collected from Decedent 3 after initiation of extracorporeal liver cross-circulation induced platelet agglutination at lower ristocetin concentrations, more efficiently than pre-extracorporeal liver cross-circulation plasma. This indicates an enhanced functional interaction between post-extracorporeal liver cross-circulation VWF and its platelet receptor, CD42 (43). We speculated that the porcine liver was synthesizing pVWF, and this was being secreted into the circulation of the decedent after connection to the extracorporeal liver cross-circulation. We evaluated VWF activity in Decedent 3 and observed that VWF activity increased after connection to extracorporeal liver cross-circulation (Figure 4B).

Because the functional assay does not distinguish between pVWF and human VWF, species-specific isoforms were quantified by mass spectrometry (MS). In all decedents analyzed, both pVWF and human VWF were detectable in the circulation. Immediately after xenograft connection, there was a pronounced surge in pVWF, reflected by an elevated pVWF-to–human VWF ratio. The pVWF-to–human VWF ratio varied over time in all decedents (Figure 4C).

Caplacizumab is an FDA-approved nanobody that specifically targets the A1 domain of VWF and blocks the ability of VWF to bind to platelets, and thereby cause their agglutination (47). We hypothesized that since plasma collected from a decedent following connection to a porcine liver had a propensity to enhance RIPA, this effect could be due to spontaneous binding of pVWF to human platelets, and if so, should be inhibited in the presence of caplacizumab.

As demonstrated in Figure 4D, caplacizumab administered ex vivo was able to effectively inhibit platelet agglutination induced by plasma from Decedent 3. However, achieving this inhibition required a higher concentration of caplacizumab when using plasma collected after exposure to the porcine liver when compared with preexposure plasma. This observation might reflect species-specific differences in the VWF, as the concentration of caplacizumab necessary to block platelet agglutination can vary depending on the species origin of the VWF (“Caplacizumab FDA Approval Package,” US FDA (2019) https://www.accessdata.fda.gov/drugsatfda_docs/nda/2019/761112Orig1s000MultiR.pdf).

To further assess whether plasma obtained from a decedent connected to a porcine liver could activate platelets, we employed a well-established flow cytometry assay (48, 49). Platelet activation was evaluated by 2 complementary markers: (a) binding of an anti–PAC-1 antibody, which specifically recognizes the activated conformation of the platelet integrin αIIbβ3 (fibrinogen receptor), and (b) detection of surface-exposed CD62P (P-selectin), a protein stored in α-granules and rapidly translocated and exposed to the platelet surface upon activation (48, 49).

As demonstrated in Figure 4E, plasma collected from Decedent 3 induced robust binding of both activation-dependent antibodies, indicating significant platelet activation under these conditions. Notably, the addition of caplacizumab to the plasma ex vivo prior to mixing with human platelets entirely abrogated anti–PAC-1 and anti-CD62P antibody binding, indicating that caplacizumab effectively inhibits post-extracorporeal liver cross-circulation porcine liver plasma-mediated platelet activation.

Collectively, these findings demonstrate that plasma collected from the decedent obtained after connection to the porcine liver contains a VWF capable of promoting robust human platelet agglutination. Furthermore, higher concentrations of caplacizumab are required to neutralize this agglutinating activity than are required to block agglutination initiated by human VWF. This indicates that VWF in the post-extracorporeal liver cross-circulation plasma possesses functional characteristics analogous to those seen in type IIb VWD. Accordingly, these findings suggest that the predominant mechanism of xenograft-induced thrombocytopenia is mediated by pVWF.

**Figure 5:**
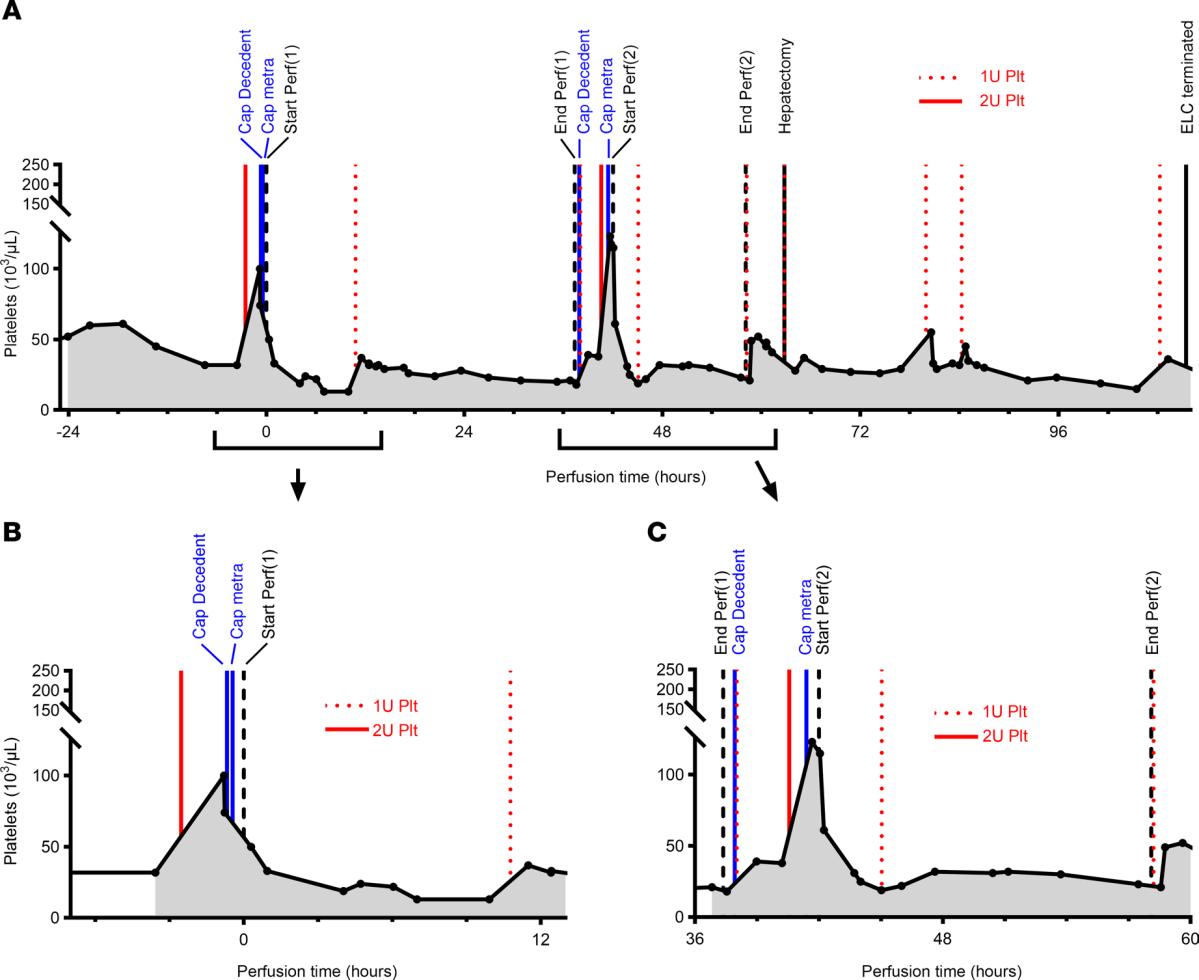
Platelet counts in the decedent receiving caplacizumab during extracorporeal liver cross-circulation with porcine livers. (**A**) Serial platelet counts of Decedent 4 before, during, and after disconnection from extracorporeal liver cross-circulation. (**B**) Expanded timeline of Decedent 4 during first porcine liver support. (**C**) Expanded timeline of Decedent 4 after first extracorporeal liver cross-circulation discontinuation, during second porcine liver support, and after second extracorporeal liver cross-circulation discontinuation before native liver hepatectomy. Red lines: infusion of platelets. Cap: infusion of caplacizumab into the decedent or the *metra* system.

Caplacizumab was evaluated as an intervention for thrombocytopenia in Decedent 4, a brain-dead recipient who exhibited marked thrombocytopenia prior to porcine liver connection, as depicted in Figure 5. Laboratory analysis revealed a substantially elevated D-dimer level of approximately 28 μg/mL, along with the presence of schistocytes and nucleated red blood cells detected on a peripheral blood smear (Supplemental Figure 1; supplemental material available online with this article; https://doi.org/10.1172/JCI200800DS1). These findings were indicative of disseminated intravascular coagulation (DIC) as the etiology of pre-extracorporeal liver cross-circulation thrombocytopenia. It should also be noted that Decedent 4 required replacement of the porcine liver after approximately 36 hours due to insufficient arterial flow.

The FDA-approved dosing regimen for thrombotic thrombocytopenic purpura begins with 11 mg of caplacizumab administered twice on the first day (totaling 22 mg), followed by 11 mg once daily. This approach is designed to maintain a plasma concentration of approximately 0.7 mg/L, exceeding the 0.5 mg/L level required to neutralize human VWF. Since we determined that higher concentrations of caplacizumab are required to neutralize this agglutinating activity of decedents connected to porcine livers (Figure 4D), we administered an amount larger than the FDA-approved dose. A 22 mg dose of caplacizumab was administered to Decendent 4 prior to initiation of extracorporeal liver cross-circulation with the first porcine liver, followed by a second 22 mg dose approximately 48 hours later before initiating extracorporeal liver cross-circulation with the second porcine liver. We also supplemented the normothermic perfusion circuit with 22 mg of caplacizumab for 1 hour prior to extracorporeal liver cross-circulation initiation on both occasions.

We observed that the magnitude of the platelet decline in Decedent 4 was substantially less pronounced than in the other decedents, as shown in Figure 5. Whereas prior experiments demonstrated a xenograft-associated platelet reduction of approximately 90%, the platelet count in Decedent 4 decreased by only approximately 50%, suggesting a potential protective effect of caplacizumab on xenograft-induced platelet consumption. However, we acknowledge that this observation occurred in the setting of brain death–associated DIC, which may confound the interpretation of this experiment and limit definitive understanding of the in vivo response to caplacizumab.

**Figure 6:**
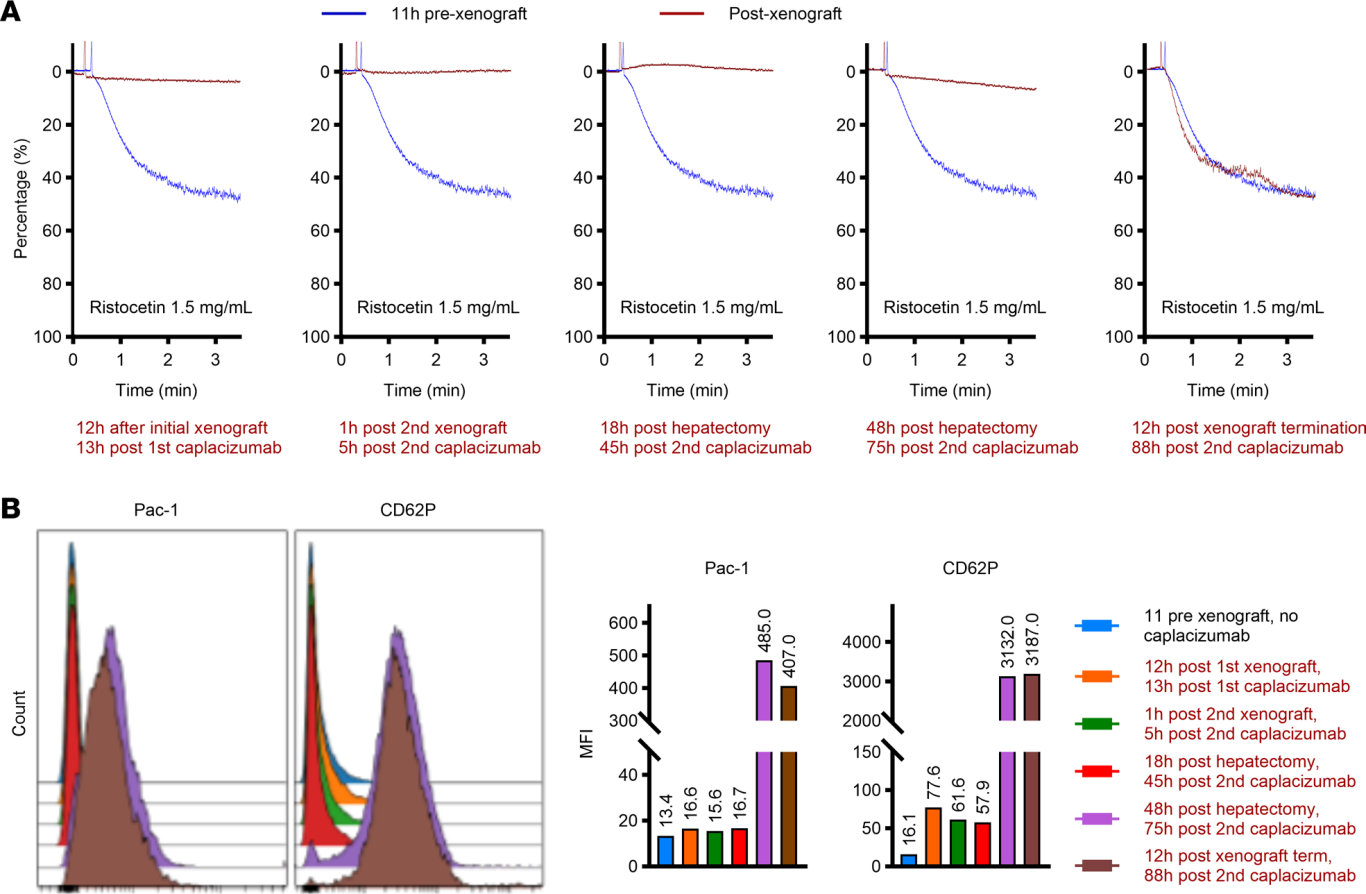
Caplacizumab blocks post-extracorporeal liver cross-circulation plasma induced platelet agglutination and activation. (**A**) Ristocetin-induced platelet agglutination (RIPA) assays are shown using plasma from caplacizumab-treated Decedent 4, obtained either before extracorporeal liver cross-circulation (blue) or at various time points after extracorporeal liver cross-circulation with a porcine liver (red). (**B**) Plasma from Decedent 4 was added ex vivo to healthy human control platelets, and platelet activation was detected by the binding of the PAC-1 (activated integrin) or the CD62P (P-selectin) antibody. Plasma obtained from the caplacizumab-treated Decedent 4 activated platelets in a pattern that depended on when the plasma was obtained relative to the start and end of caplacizumab therapy.

We next evaluated whether caplacizumab administration in Decedent 4 mitigated the ex vivo platelet abnormalities previously identified. Caplacizumab administration to Decedent 4 completely suppressed platelet-induced agglutination in the modified ex vivo RIPA assay (Figure 6A). This result demonstrates that in vivo administration of caplacizumab reversed the xenograft-induced platelet agglutination defects observed ex vivo.

In addition, plasma obtained from the caplacizumab-treated Decedent 4 did not induce platelet activation, as evidenced by the absence of PAC-1 and CD62P antibody binding at all early time points following xenograft connection (Figures 6B). However, it is important to note that plasma obtained 75 hours after the last in vivo dose of caplacizumab (corresponding to approximately 4 elimination half-lives) was once again capable of inducing platelet activation in the flow cytometry assay, consistent with a time-dependent loss of inhibitory activity as circulating caplacizumab concentrations waned.

## Discussion

**Figure 7:**
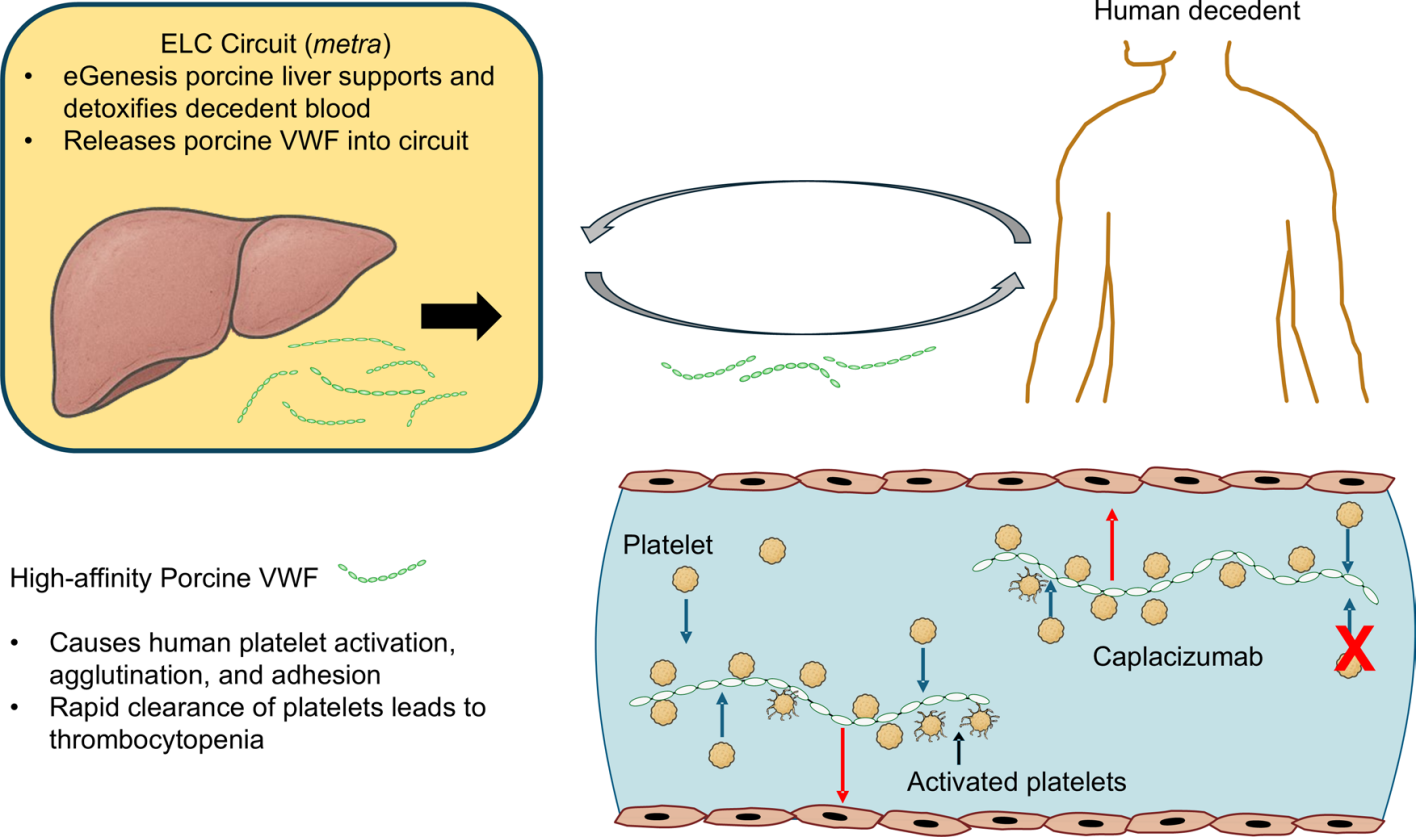
Mechanistic model of liver xenograft induction of thrombocytopenia. Schematic representation showing that genetically modified porcine liver xenografts restore hepatic detoxification and protein synthesis but trigger rapid human platelet clearance through spontaneous binding of porcine von Willebrand factor (pVWF) to human GPIbα (CD42). This is analogous to type IIb von Willebrand disease. Recipient plasma–induced platelet activation is inhibited ex vivo or in vivo by the VWF antagonist caplacizumab, identifying pVWF-mediated activation as the principal mechanism of xenograft-associated thrombocytopenia. ELC, extracorporeal liver cross-circulation.

Hematologic dysregulation appears to be the dominant unresolved barrier to successful liver xenotransplantation, as porcine liver grafts consistently provoke rapid and profound thrombocytopenia even when immunologic rejection is well controlled (19, 25, 31, 50). In this study, extracorporeal cross-circulation with a transgenic porcine liver in brain-dead human recipients caused uniform, severe platelet loss (affecting both endogenous and transfused platelets) within the first hour. Ex vivo assays demonstrated that post–cross-circulation plasma uniquely induces platelet agglutination and activation. These effects were blocked by caplacizumab (an anti-VWF nanobody), highlighting pVWF-platelet interactions as a key therapeutic target. The data support a model in which structurally distinct pVWF, analogous to the type IIb VWD phenotype, drives spontaneous platelet adhesion, agglutination, and aggregation, leading to accelerated platelet clearance (Figure 7). Previous work decades ago by Meyer and colleagues showed that a sequence difference in the pVWF A1 domain likely explains the spontaneous binding to its receptor on human platelets (51).

Work in nonhuman primates has suggested that pVWF may contribute to xenograft-induced thrombocytopenia. Studies by Gaca et al. and LaMattina et al. demonstrated that administration of an antibody blocking pVWF-platelet interactions attenuated both thrombocytopenia and DIC in baboon recipients of porcine lung xenografts (44, 45). Building on these findings, Connolly et al. showed that genetic modification of the pVWF gene could partially prevent thrombocytopenia in a nonhuman primate model of porcine lung xenotransplantation (46). In contrast, Zheng et al. reported a case in which transplantation of a porcine liver as an auxiliary graft in a human recipient led to thrombocytopenia attributed to complement-mediated platelet destruction (13). However, this interpretation is complicated by the rapid resolution of thrombocytopenia following simultaneous anti-complement therapy and removal of the xenograft.

It is notable that higher concentrations of caplacizumab are required to bind to and neutralize pVWF as compared with human VWF. This observation demonstrates species-specific pharmacodynamics, and suggests that compared with human VWF, pVWF has a higher binding affinity for human platelets. These findings raise the possibility that engineering targeted therapeutics analogous to caplacizumab, but specifically directed against pVWF, could offer a more potent and selective strategy to block pathologic platelet binding. However, for such therapies to be viable, significant suppression of antibody-mediated responses will also be essential, since porcine coagulation factors themselves may be immunogenic. This risk is underscored by prior clinical observations where porcine factor VIII, when used to treat acquired hemophilia, elicited robust anti-porcine xenoantibody responses in human recipients (52).

This work raises the question of what is the best therapeutic strategy to prevent xenotransplant-induced thrombocytopenia. Our data demonstrate that the porcine liver secretes pVWF that spontaneously binds to human platelets and induces activation and agglutination. Caplacizumab, by blocking the VWF-platelet interaction, can mitigate, but not entirely prevent, thrombocytopenia, even when the dose is increased to account for the greater functional potency of pVWF. Our attempt to mitigate the thrombocytopenia by administering caplacizumab in Decedent 4 was completely effective at impairing ex vivo platelet activation. The optimal dosing strategy to prevent thrombocytopenia in vivo in the decedent model still needs to be established. Our assessment of the effect of caplacizumab on thrombocytopenia in vivo may have been confounded by the presence of DIC in Decedent 4, which independently influenced platelet kinetics. However, caplacizumab efficacy wanes, as observed in plasma samples after the final dose, indicating the need for sustained or higher dosing regimens. Administering desmopressin (DDAVP) prior to connecting the porcine liver to extracorporeal liver cross-circulation may transiently deplete its presynthesized stores of pVWF, although prior ex vivo studies in a pulmonary xenotransplant model showed only a limited benefit with this approach (53). Alternatively, genetic modification of the porcine liver to “humanize” the pVWF and reduce pVWF-platelet interactions may offer a potential strategy to mitigate the thrombocytopenia. This genetic approach has been employed with some success in porcine lung xenotransplants into a nonhuman primate model (46). In addition, tailored biologics such as an anti-pVWF therapeutic may offer a more potent and selective means to inhibit porcine liver–induced platelet activation, which could complement dosing strategies and genetic modifications. Extending these findings into early-phase hepatic xenograft clinical studies could yield valuable insights.

Limitations of this study include the small sample size of 4 decedents, which constrains generalizability, and the limited duration of porcine liver support, which precludes assessment of longer-term hematologic and immunologic effects. Our use of a brain-dead decedent model also introduces important interpretive limitations. Brain death is associated with autonomic dysregulation, systemic immune activation, and activates coagulation pathways and platelet function — all potentially complicating factors that could contribute to the thrombocytopenia in the decedents (54, 55). However, the immediacy of thrombocytopenia after xenograft connection makes a de novo adaptive immune response to the porcine liver unlikely because such an immune or inflammatory mechanism should already have been operative in the proinflammatory milieu of brain death before perfusion. In vivo administration of caplacizumab to Decedent 4 appeared to blunt the severity of thrombocytopenia, but concomitant DIC in this recipient limits definitive interpretation of the treatment effect. Accordingly, therapeutic strategies such as anti-VWF agents require evaluation in longer-term survival models.

In the future, porcine liver xenotransplantation may require a multipronged strategy combining advanced genetic modifications, targeted biologic therapies, and intensive immunomodulation. Humanizing pVWF and other key coagulation factors through precise genome editing, while simultaneously developing therapeutics such as species-tailored anti-VWF antibodies, could help minimize platelet activation and clearance. Anticipating and suppressing anti-xenoantibody responses against newly synthesized porcine coagulation proteins might be important for preventing both acute and delayed graft dysfunction. Ultimately, an integrated approach, validated through rigorous early-phase human trials, will be essential to achieve safe, reliable, and durable hepatic xenograft function in clinical settings.

## Methods

### Sex as a biological variable.

Sex was not considered as a biological variable throughout the entire study.

### Decedent selection and management.

Four brain-dead donors (2 male, 2 female; ages 57–80 years) were enrolled in the study conducted at the Gift of Life Donor Program (GLDP) Donor Care Center at the Hospital of the University of Pennsylvania, where comprehensive critical care support was available. All decedents were declared brain dead and considered for enrollment only after all efforts to place transplantable organs had been exhausted. Decedents 2 and 3 developed acute-on-chronic kidney dysfunction prior to extracorporeal liver cross-circulation initiation and received continuous renal replacement therapy throughout the study. Pretrial surgical exploration in Decedent 3 revealed underlying liver cirrhosis.

### Liver xenograft source and preparation.

All EGEN-5784 livers were provided by eGenesis. They were procured from ESUS-1784 Yucatan minipig donors with the following genetic modifications: (a) functional knockout of genes for glycan-producing enzymes α-1,3-galactosyltransferase (*GGTA1*), cytidine monophosphate-N-acetylneuraminic acid hydroxylase (*CMAH*), and β-1,4-N-acetylgalactosaminyltransferase 2/-like (*B4GLANT2/B4GLANT2L*) to prevent hyperacute rejection; (b) 7 human transgene insertions (*PROCR*, *THBD*, *TNFAIP3*, *HMOX1*, *CD46*, *CD47*, and *CD55*) to improve coagulation, complement, and immune regulation compatibility between species; and (3) inactivation of the *pol* gene to inactivate porcine endogenous retroviruses. All donor pigs tested negative for porcine cytomegalovirus, porcine lymphotropic herpesvirus, porcine circovirus 3, and hepatitis E virus.

### Normothermic porcine liver perfusion and extracorporeal liver cross-circulation.

EGEN-5784 livers underwent initial isolated perfusion using the *metra* system (OrganOx), a clinically validated normothermic liver perfusion device extensively primed with human red blood cells and albumin. The system was then connected to the femoral and internal jugular veins of the decedents via extension cannulas using a bespoke exchange system (*metra*-extracorporeal liver cross-circulation, OrganOx), maintaining continuous exchange at 0.4 L/min. This setup allowed for real-time visualization of the extracorporeal liver and facilitated scheduled liver biopsies. Heparin infusion was administered throughout extracorporeal liver cross-circulation to maintain an activated clotting time of 160–200 seconds.

### Decedent management and surgical interventions.

Extracorporeal liver cross-circulation was performed for 72 hours in Decedents 1 and 3, 84 hours in Decedent 2, and 114 hours in Decedent 4. Extracorporeal liver cross-circulation was electively terminated in Decedents 1, 2, and 3. Clinical management concluded with cessation of circulation in Decedent 1 at 72 hours, while Decedents 2, 3, and 4 remained under critical care observation for an additional 17–36 hours following extracorporeal liver cross-circulation discontinuation. Decedent 4 underwent a total hepatectomy of the native liver with creation of a portocaval shunt at 63 hours after extracorporeal liver cross-circulation initiation and was supported exclusively by the liver xenograft for 48 hours before extracorporeal liver cross-circulation was electively discontinued. The decedent remained anhepatic for an additional 18 hours before developing cardiac arrest. Decedent 2 received IVIg, Decedent 3 received pegcetacoplan, and Decedent 4 received caplacizumab, as detailed in the Results section.

### Immunosuppression and anticoagulation.

All decedents received daily intravenous methylprednisolone (500 mg) as the sole immunosuppressive therapy throughout the study period. Since all decedents received heparin, protamine was added to all blood samples before analysis.

### Platelet adhesion to endothelial cell assay.

Static assay: Porcine primary vein endothelial cells (Cell Biologic, P-6009) or human umbilical vein endothelial cells (HUVECs, Lifeline Cell Technology) were cultured in a 24-well plate until greater than 90% confluent. Washed platelets (1 × 10^7^) derived from a healthy donor were plated on the endothelial cell layer and incubated at 37°C for 30 minutes. Nonadherent cells were collected and counted to calculate the percentage of platelet adherence. Caplacizumab (1 μg/mL) was used to specifically block the VWF-mediated platelet adhesion.

### Microfluidic assay.

A total of 6 × 10^6^ cells/channel of porcine primary vein endothelial cells or HUVECs were seeded into fibronectin-coated (50 μg/mL, Sigma-Aldrich, F0895) channels of a 48-well microfluidic plate (Bioflux, Fluxion Biosciences) (56). Two hundred microliters of platelet suspension (2 × 10^6^ platelets in HBSS with Ca^2+^/Mg^2+^) from healthy donors was labeled with calcein-AM (2 μg/mL final concentration; Thermo Fisher Scientific, C3100MP) for 15 minutes just prior to being flowed through the channels at 10 dynes/cm^2^. Platelet accumulation on the endothelium field was captured over the next 15 minutes by a Zeiss Axio Observer Z1 inverted microscope using Montage Fluxion software and analyzed using ImageJ, as previously described (56). Caplacizumab (1 μg/mL) was used to specifically block the VWF-mediated platelet adhesion.

### Platelet agglutination/aggregation.

Platelets isolated from healthy donors were resuspended in HEPES-Tyrode’s buffer. Washed platelets (5 × 10^7^ platelets/mL) supplemented with 1 mM CaCl_2_ were added to decedent plasma samples in the presence of ristocetin (Chrono-Log, P/N 396) to induce the agglutination/aggregation. Caplacizumab-yhdp (CABLIVI) was preincubated with the reaction mixture to block the RIPA. The agglutination/aggregation was measured by the turbidometric method at 37°C in a Lumi-Dual aggregometer (Chrono-Log, Model 700).

### Analysis of VWF levels in plasma samples.

Functional VWF levels were analyzed in Decedent 3 using a commercial assay (BioData Corp, 103025). For MS analysis of VWF, post-extracorporeal liver cross-circulation plasma was randomized across two 96-well plates and processed on an SP100 Automation Instrument using 2 nanoparticle suspensions from the Proteograph XT Assay Kit (Seer, Inc.). Dried samples were reconstituted (50 ng/μL) and 8 μL was loaded onto an Acclaim PepMap 100 C18 trap column (Thermo Fisher Scientific) connected to an Ultimate 3000 HPLC System (Thermo Fisher Scientific). Peptides were separated on a 50 cm μPAC HPLC column (Thermo Fisher Scientific) at 1 μL/min using a gradient of 5–25% solvent B (0.1% Formic Acid in acetonitrile) in solvent A (0.1% Formic Acid in water) over 22 minutes (37 minutes total). MS analysis was performed on an Orbitrap Astral in DIA mode (*m*/*z* 380–980, 3 *m*/*z* windows), with MS1 at 240k and MS2 at 80k resolution. Raw files were analyzed with the Proteograph Analysis Suite (Seer, Inc) using DIA-NN 1.8.1 in a library-free search with cloud Match Between Run (MBR). Three search strategies were compared: combined pig and human FASTA, and separate FASTA searches for *Sus scrofa* v11.1 and *Homo sapiens* v2023.11.09, all at 1% FDR. Nanoparticle-level PSM identifications were exported for downstream analysis.

### Flow cytometry of platelets.

For ex vivo activation assays of control platelets with decedent plasma, healthy platelets were first isolated. Whole blood collected in citrate tubes was spun at 125*g* for 15 minutes and the supernatant was collected to isolate platelet-rich plasma (PRP). PRP was subsequently spun at 330*g* for 10 minutes to collect pelleted platelets that were resuspended in 1× Tyrode’s buffer. Platelets were subsequently activated on a 96-well plate with 50 μL of isolated plasma derived from decedent patients for 30 minutes. The plasma-mediated activation was terminated by adding PBS/4% paraformaldehyde for 20 minutes. The platelets were stained with anti–human CD42b (BD Biosciences, 551061), anti–human CD62P (BD Biosciences, 555524), and anti–human CD41/61 Pac-1 (BD Biosciences, 340507).

### Histology.

Staining of liver sections was performed by Applied Pathology Systems. Briefly, sections (5 μm) of porcine liver were baked at 60°C for 1 hour, rehydrated, and subjected to Tris-EDTA (pH 9.0) antigen retrieval by pressure cooking at 102°C for 30 seconds. After blocking with Bloxall and 5% horse serum, sections were incubated for 1 hour at room temperature with an anti-CD41 polyclonal antibody (Proteintech, 24552-1-AP; 1:2000). Slides were then processed with goat anti-rabbit amplifier and horse anti-goat–HRP, developed with DAB, counterstained with hematoxylin, dehydrated, and coverslipped and analyzed by a board-certified anatomic pathologist and a board-certified hematologist.

Blood cell morphology was analyzed on Wright-Giemsa staining of peripheral blood smears and reviewed independently by 2 board-certified hematologists. Alternatively, blood cells were analyzed using a CellaVision analyzer that captured digital images of the blood smear, which were then analyzed by the software to provide a preclassification that board certified hematologists reviewed and verified.

### Statistics.

Two-way ANOVA with Tukey’s test for multiple comparisons was used to compare the adhesion in Figure 3B. A *P* value less of than 0.05 was considered statistical significance.

### Study approval.

Study approval was obtained from the board and leadership of the GLDP (Philadelphia, Pennsylvania, USA), and the protocol was reviewed and approved by a study-specific Medical Oversight Committee convened by eGenesis (Cambridge, Massachusetts, USA). Written authorization was provided by the next-of-kin of potential decedents, permitting extracorporeal liver cross-circulation for up to 72 hours, with selective extension to 7 days as needed.

### Data availability.

Quantitative data underlying Figure 3B, including all individual data points shown in the graph, are provided as a Supporting Data Values file. All other data are available from the corresponding author upon request.

## Author contributions

LZ, SAA, A Shaked, QG and CSA designed research studies, conducted experiments, acquired and analyzed data, and wrote the manuscript. A Suzuki and EEF analyzed data and wrote the manuscript. A Sarkar designed research studies, conducted experiments, and acquired and analyzed data. A Sagar conducted experiments, acquired data, and wrote the manuscript. FL, JF, MAE, and SHA conducted experiments. LL, KG, and SCL designed research studies and wrote the manuscript. KMO designed research studies. BJK, PF, and MP designed research studies, analyzed data, and wrote the manuscript.

## Conflict of interest

SHA, MAE, JF, and A Sagar are consultants for OrganOx. PF is co-founder and Chief Medical Officer of OrganOx. KG, LL, and SCL are employees of eGenesis. KMO is on the Advisory Board for OrganOx.

## Funding support

This work is the result of NIH funding and is subject to the NIH Public Access Policy. Through acceptance of this federal funding, the NIH has been given a right to make the work publicly available in PubMed Central.

- NIH grants HL146373 and HL148014 (to CSA).
- NIH grants HL150698 and R35HL150698 (to MP).
- NIH grants AI191397 and U01AI191397 (to A Shaked).
- NIH grant K08-AR081929 (to SAA).
- Hemostasis and Thrombosis Research Society grant GRT-00005101 (to A Sarkar).
- Rheumatology Research Foundation Scientist Development Award (to SAA).
- Arthritis National Research Foundation (to SAA).

## Supplementary Material

Supplemental data

ICMJE disclosure forms

Supporting data values

## Version 1 03/10/2026

In-Press Preview

## Version 2 05/01/2026

Electronic publication

## Version 3 05/04/2026

Corrected duplicate label on Figure 4D
