# Supplementary material for: Graft-derived VWF drives platelet activation and thrombocytopenia during porcine liver xenotransplantation to brain-dead human recipients
Source: J Clin Invest. 2026 Mar 10;136(9):e200800. doi: 10.1172/JCI200800 (PMC13132372; doi:10.1172/JCI200800)
Supplement: Supplemental data [file jci-136-200800-s008.pdf]

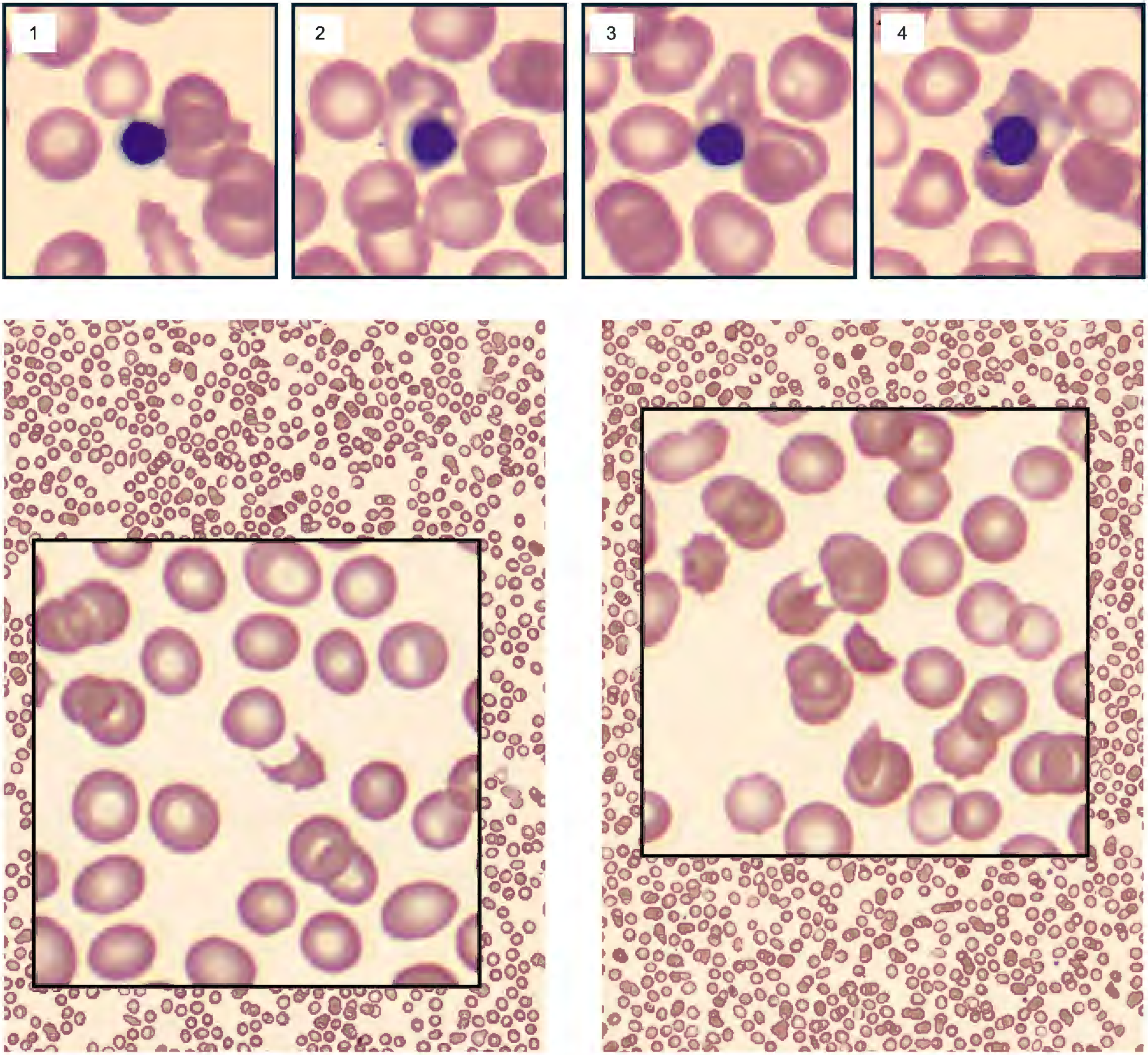
1
2
3
4

Supplemental Figure 1: Disseminated Intravascular Coagulation in Decedent 4 prior to exposure to the porcine liver. Shown are Cellavision Digital Capture images from a peripheral blood smear of decedent prior to connection to extracorporeal liver cross-circulation with the porcine liver. Schistocytes and nucleated red blood cells were easily seen. The D-Dimer level was 27.72 ug/ml.
